# Practical Approaches to Build and Sustain a Cardio-Oncology Clinic

**DOI:** 10.3390/jcdd9050158

**Published:** 2022-05-14

**Authors:** Angeliki Chasouraki, Christos Kourek, Alexandros Sianis, Konstantinos Loritis, Peggy Kostakou, Elias Tsougos, Ioannis Paraskevaidis, Argyrios Ntalianis, Alexandros Briasoulis

**Affiliations:** 1 Department of Clinical Therapeutics, National Kapodestrian University of Athens, 11528 Athens, Greece; angchasouraki@gmail.com (A.C.); chris.kourek.92@gmail.com (C.K.); sianisalex@gmail.com (A.S.); lork21994@gmail.com (K.L.); pkostakou@gmail.com (P.K.); itsougos@hygeia.gr (E.T.); iparask@otenet.gr (I.P.); arg_nt@yahoo.gr (A.N.); 2 Division of Cardiovascular Diseases, University of Iowa Hospitals and Clinics, Iowa City, IA 52242, USA

**Keywords:** cardiotoxicity, heart failure, chemotherapy, cardio-oncology

## Abstract

The therapeutical advances in recent years in the field of oncology treatment have increased survival rates and improved the quality of life of oncology patients, thus turning cancer into a chronic disease. However, most of the new cancer treatments come at the expense of serious cardiovascular adverse events threatening the success story of these patients. The establishment of multidisciplinary medical teams to prevent, monitor, and treat cardiovascular diseases in cancer-treated patients is needed now more than ever. The aim of this narrative review is to demonstrate the existing knowledge and practical approaches on how to establish and maintain a cardio-oncology program for the rising number of patients who need it.

## 1. Introduction

Cardiotoxicity is a significant adverse reaction to chemotherapy and radiotherapy, leading to increased morbidity and mortality and a poor prognosis and quality of life of cancer patients [1,2]. Cardiotoxicity presents various clinical subtypes, ranging from asymptomatic structural changes in cardiac imaging techniques or subclinical arrhythmias in echocardiogram (ECG) to serious clinical symptoms that require urgent hospital admission and can lead to irreversible heart failure or even death [2]. The onset of cardiotoxicity is unknown, and thus it could be developed early during chemotherapy, later within the first year after chemotherapy, or very late after one year of chemotherapy and sometimes even one decade after cancer chemotherapy completion [2,3]. The most common chemotherapy drugs related to cardiotoxicity include 5-fluorouracil, paclitaxel, anthracyclines, and targeted therapies such as monoclonal antibodies and tyrosine kinase inhibitors [2,3,4,5,6,7].

Establishing a cardio-oncology program to prevent, monitor, and treat cardiovascular adverse events in patients with cancer after chemotherapy and/or radiotherapy is one of the most challenging fields of medicine nowadays. A cardio-oncology team should consist of experienced cardiologists, hemato-oncologists, oncologists, nurses, and operational staff who collaborate to provide comprehensive cardiovascular care to oncology patients in a community hospital or in an outpatient department [8,9,10]. Baseline risk stratification prior to receiving cancer therapies and monitoring during but also after chemotherapy/radiotherapy completion and interventions in order to prevent cardiotoxicity are the basic principles for the sustainability of a cardio-oncology program.

The aim of the present review is to demonstrate the existing knowledge and practical approaches on the ways a cardio-oncology program could be built and sustained for patients, either before they develop or after establishing cardiotoxicity due to cancer therapy.

## 2. Cardio-Oncology Team Members

The purpose of a cardio-oncology clinic is to assess and monitor oncology patients under chemotherapy and/or radiotherapy with cardiovascular adverse events. A multidisciplinary approach is required in order to achieve this goal. Cardiologists, medical and radiation oncologists, hematologists, and nurses usually form the multidisciplinary team of the program collaborate and meet regularly to make decisions regarding treatment in cardio-oncology patients [11]. Family doctors, internal medicine physicians, and pharmacists can also provide helpful guidance to the team [12]. The role of the palliative care team, in co-operation with psychologists, is to attend to the patient’s daily physical and mental health and support their family when it is necessary. Another significant role of the cardio-oncology program is the administrative staff who co-ordinates the multidisciplinary team, organizes patient follow-up appointments and tests, and updates the registry base with further information and laboratory and imaging tests. The registry base should be able to provide all the necessary information to the multidisciplinary team in order to reach a safe strategy. The registry base should include patient demographics, physician contact information, previous medical history, cardiovascular risk factors, details about malignancy (TNM, prognosis), previous and ongoing oncology treatment (dosages, timing), laboratory and imaging results of the baseline cardiovascular evaluation, risk stratification, scheduled follow-up appointments, and medication list [11]. Another important factor of the cardio-oncology care that ensures a well-informed care and patient adherence to the program is the continuous education of both the patients and staff [12]. Cardio-oncology training of the healthcare members of the team and educational procedures, such as through healthcare staff meetings, seminars, and conferences and by providing pamphlets to patients, are very important [12,13]. Once the cardio-oncology unit is set up, the multidisciplinary team could arrange a meeting to evaluate high-risk patients and make treatment decisions, thus providing high-quality care [11].

The development, infrastructure, and challenges related to cardio-oncology clinics in Greece are described in the following published manuscript ‘Taskforce of the Hellenic Heart Failure Clinics Network’ [14].

## 3. Services of a Cardio-Oncology Program

**Figure 1:**
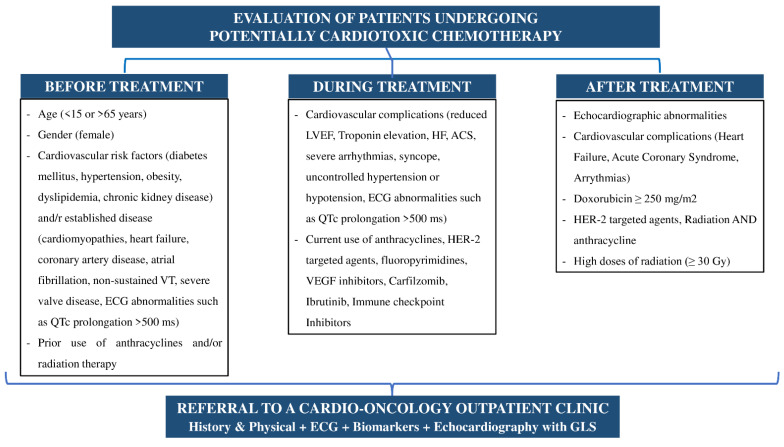
Suggested evaluation of patients with malignancies at risk for cardiotoxicity. (Figure 1 is an original figure, also including data from reference [21]).

**Figure 2:**
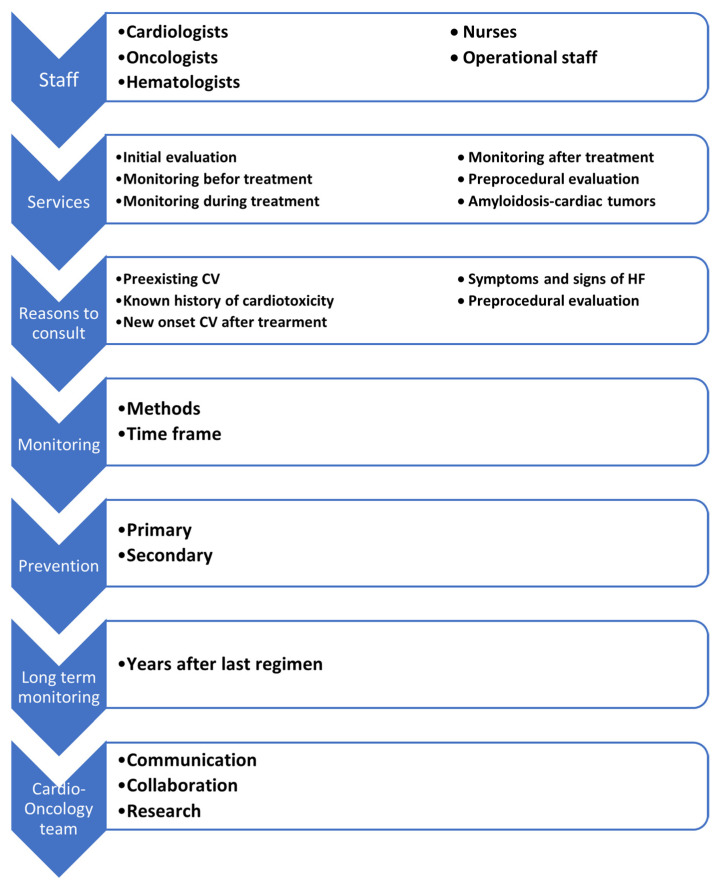
Steps in planning and developing a cardio-oncology clinic.

Cardiotoxicity caused by chemotherapeutics and/or radiotherapy is a common complication in patients with cancer. It may occur in up to 25% of patients with breast cancer [15]. The underlying cardiovascular risk factors, the chemotherapeutic agent, and the amount of radiation administered are the most crucial factors that determine this percentage [15]. As the incidence rates of cardiotoxic complications, including arrhythmias, heart failure, myocarditis, pericardial effusion, myocardial ischemia, coronary artery disease, arterial and pulmonary hypertension, valvular diseases, and cardiac-related mortality, are mostly associated with anthracyclines (1–26%) [15,16,17], high-dose cyclophosphamides (7–28%) [15,17,18,19], trastuzamab (2–28%), and tyrosine kinase inhibitors (0.05–11%) [15,17], all patients with cancer should be referred to a cardio-oncology outpatient department immediately after the start of chemotherapy and evaluated by an expert cardio-oncology team [12,20]. There are different stages of evaluation for cancer patients. More specifically, there are some crucial time points [21]:

i. The first time point is immediately after the diagnosis of the disease and before any treatment is administered. The purpose of this stage is the initial risk stratification for cardiotoxicity and the possibility of a potential cardiac monitoring and/or treatment [21].
ii. The second time point is during chemotherapy at regular intervals depending on the type of treatment and especially when heart failure symptoms or signs appear [21].
iii. The last, but not least important timepoint is after the end of treatment with chemotherapeutics and/or radiation, especially in patients who have experienced cardiotoxicity or are a candidate to develop long-term complications of the cardiovascular system [21]. The evaluation of patients with cancer and steps of the development of the cardio-oncology clinic are summarized in Figure 1 and Figure 2.

The most important stage of the above is the first one where the initial risk stratification for cardiotoxicity is performed. An extensive medical history, including clinical examination, ECG, laboratory exams, and imaging techniques, such as chest X-rays and cardiac ultrasounds, is required in order to classify patients as being either at a low or high risk for developing cardiac complications due to cardiotoxicity after cancer treatment [12]. Based on the initial risk stratification, modifications to the type of chemotherapy, the radiation quantity, or the existing therapy for cardiovascular disease, including coronary artery disease, hypertension, or heart failure, may be necessary. In case there is a solid tumor cancer that needs to be removed, a pre-operative assessment is also available by the cardio-oncology team [12,20,21].

A cardio-oncology team usually consists of a specialized cardiologist, a hematologist, and an oncologist [12]. This multispecialty approach increases the coordination, communication, and collaboration among healthcare members and improves the overall care, quality of life, and clinical outcomes of cancer patients [12]. Other factors, including facilities, technological equipment, and experienced staff, improve the possibilities of the cardio-oncology program even further.

In summary, the services provided by a cardio-oncology program are the evaluation of patients with cancer before, during, and after chemotherapy; the risk stratification for developing cardiotoxicity due to chemotherapeutic agents; the pre-operative assessment in solid tumor cancers that need to be removed; and monitoring or treating special subgroups of patients with cancer that are directly associated with the cardiovascular system, such as amyloidosis and cardiac tumors.

## 4. Patient Referral Criteria

It is not always feasible for the cardio-oncology clinics to evaluate every patient with cancer who is a candidate to undergo chemotherapy. A possible explanation could be the fact that there is a large number of patients, while cardio- oncology departments are limited. Another reason is the low risk of most patients in presenting cardiotoxicity. Therefore, it would be reasonable to establish referral criteria for these patients so that high-risk patients could have priority and, thus, greater benefit from their evaluation by the cardio-oncology team [12,20,21]. These criteria include:

i. Medical history of established cardiovascular disease or multiple (two or more) cardiovascular risk factors.
ii. History of previous cardiotoxicity, receiving a potentially cardiotoxic chemotherapy, or history of previous radiotherapy in the chest area.
iii. Onset of symptoms or signs of heart failure before, during, or after cancer treatment.
iv. Special priority should be given to a pre-operative evaluation of patients with solid tumor cancer who are candidates to undergo surgery or patients with types of cancer that directly affect the cardiovascular system (amyloidosis and cardiac tumors).

All the above criteria need to be determined in agreement with all members of the cardio-oncology units in order to optimize the provided services. Actions that could result in the establishment of acceptable referral criteria are the creation of referral forms for oncology patients to cardio-oncology units and the development of electronic health records. This proposal would significantly help in referring the right patients, securing the necessary information from their medical history, and improving communication between physicians.

## 5. Follow-Up Protocols

One of the most difficult steps toward the operation of the cardio-oncology unit is the establishment of follow-up strategies for oncology patients, since data are still limited [22]. All patients should undergo a cardiovascular evaluation prior to starting chemotherapy treatment that could lead to cardiotoxicity in order to establish a baseline cardiovascular (CV) risk for the upcoming treatment and follow-up. The initial patient assessment should include previous medical history and cardiovascular risk factors, physical examination focused on the cardiovascular system, previous cancer therapy history, blood pressure measurement, ECG, echocardiogram, and laboratory tests. Risk factors that should be considered are lifestyle, including smoking habits and BMI; CV risk factors, including hypertension, diabetes, dyslipidemia, and chronic kidney disease; and previous CV disease. Laboratory tests should include lipidemic profile, HbA1c, and cardiac biomarkers, such as troponin and natriuretic peptides [23]. Further imaging examinations, such as stress echo or 24 h Holter, are indicated for certain patients [22].

Chemotherapy treatment is associated with certain cardiovascular complications. Type 1 cardiotoxicity is irreversible and dose-related, while type 2 cardiotoxicity is reversible but not dose-related. Type 1 cardiotoxicity was firstly defined as toxicity by anthracyclines, whereas type 2 cardiotoxicity was defined as toxicity by trastuzumab. Another definition was based on whether the myocardial damage was reversible or not. Specifically, type 1 toxicity was considered to be permanent and irreversible (myocardial damage), while type 2 toxicity was mainly reversible within 2–4 months after chemotherapy (myocardial dysfunction). Anthracyclines are characteristic agents that may cause type 1 cardiotoxicity presented as heart failure, asymptomatic left ventricular systolic dysfunction, and/or arrhythmias. Agents including human epidermal growth factor receptor-2 (HER-2)-targeted therapies, vascular endothelial growth factor (VEGF) inhibitors, immunotherapy checkpoint inhibitors (ICIs), and proteasome inhibitors may cause type 2 cardiotoxicity that usually presents as heart failure, left ventricular systolic dysfunction (LVSD), arrhythmias, hypertension, or myocardial ischemia [8]. Radiation-induced cardiac injury presents as myocarditis, pericarditis, restrictive cardiomyopathy, constrictive pericarditis, coronary artery disease, valvular diseases, or conduction disturbances. Occasionally, radiotherapy can be related to the calcification of the thoracic aorta (porcelain aorta) or other big vessels [24]. Myocarditis caused by immune checkpoint inhibitors is a separate type of myocardial damage, related to immune phenomena and should be viewed and managed separately, not in the spectrum of established types of cardiotoxicity.

During the initial evaluation, patients are usually subcategorized as low, intermediate, high, and very high-risk patients. In cases of previous anticancer therapies with high-dose anthracycline, high-dose radiotherapy, or low-dose anthracycline combined with low-dose radiotherapy, patients are at a higher risk of developing cardiac adverse events. Patients who receive low doses of anthracycline or trastuzumab accompanied by more than two CV risk factors, those who receive trastuzumab with a previous history of low-dose anthracycline, more elderly patients, and patients with cardiac dysfunction are also characterized as high-risk [23]. According to the Mayo Clinic, patients scheduled to receive anthracyclines, trastuzumab, cyclophosphamide, ifosfamide, or clofarabine are at a high risk of developing cardiotoxicity [13].

There is a different score for each risk factor, which is added to the total risk. Previous heart failure or a history of cardiomyopathy accounts as very high-risk for all possible cardiotoxic agents. A history of cardiotoxicity under trastuzumab therapy could entail a very high risk for patients who are scheduled to receive HER-2-targeted agents. Patients who are going to receive VEGF inhibitors are at very high risk of cardiotoxicity if they have arterial vascular disease. Cardiac amyloidosis, venous thrombosis, arterial vascular disease, or a previous history of cardiotoxicity with proteasome inhibitors are considered as very high-risk factors for patients planned to undergo proteasome inhibitor therapy [8].

There are various follow-up protocols depending on the initial cardiovascular toxicity risk and the course of cancer treatment that will be administered [22]. For example, in patients with a high risk who are about to receive anthracyclines, after the initial assessment, a 3-month re-evaluation via echocardiography is recommended for the first year, every 6 months for four years, and then annually [22]. According to the total risk stratification score, patients at an intermediate risk of cardiotoxicity should be monitored via echocardiogram with global longitudinal strain (GLS) halfway through, at the end, and 3–6 months after the completion of the treatment. High-risk patients should undergo an echocardiogram with GLS every three cycles, after the completion of the treatment, at 3–6 months, and 1 year later, whereas very high-risk patients should also be monitored before every chemotherapy cycle [13]. Follow-up protocols should be adjusted according to patient needs and circumstances in collaboration with oncologists and cardiologists [23].

Echocardiography is the recommended method for monitoring cancer-treated patients. The evaluation should include precise left ventricular ejection fraction (LVEF) measurements with the best available method, preferably 3D evaluation, and an evaluation of the cardiac valves and pericardium [25].

Monitoring cardiac biomarkers, such as troponin I, may also be a useful follow-up tool. Studies have shown that patients with positive troponin I at any time before or during treatment may have a higher incidence of cardiac dysfunction [26,27]. Positive troponin I could independently predict cardiotoxicity (*p* < 0.001). In a previous study, trastuzumab-treated patients with a positive baseline troponin I did not recover from cardiotoxicity, while patients who suffered from cardiotoxicity but never had positive troponin I recovered fully [26].

After the completion of anticancer treatment, if the patient presents with echocardiographic abnormalities or cardiovascular complications, such as heart failure or acute coronary syndrome, a referral to the cardio-oncology clinic is of high importance. Previous therapy with HER-2-targeted agents, doxorubicin ≥250 mg/m^2^, radiation, and anthracycline or high doses of radiation (≥30 Gy) indicate after-treatment evaluation [21].

## 6. Prevention Strategies

Primary prevention strategies of cardiotoxicity in patients planned to receive cardiotoxic therapy include the diagnosis and treatment of possible underlying cardiovascular disease, identification and optimal control of cardiovascular risk factors, and proper planning of anticancer therapy (e.g., optimum total dose of anthracyclines, optimal radiation chest fields, etc.). Secondary prevention strategies are implemented once clinical or subclinical cardiotoxicity is detected, including chemotherapeutic treatment modification and a possible implementation of effective therapeutic regimens and specific exercise protocols along with close supervision [22].

Dexrazoxane is an iron chelating agent that contributes to the reduction of free radicals during anthracycline therapy [28]. In a previous trial, patients treated with trastuzumab were subcategorized according to anthracycline use or not and were randomized to receive carvedilol, lisinopril, or placebo. Patients on active prevention had significantly fewer cancer treatment interruptions. On anthracycline, a higher cardiotoxicity-free survival was observed under carvedilol or lisinopril treatment [29]. The OVERCOME trial showed stable LVEF in patients receiving enalapril and carvedilol, whereas LVEF dropped in the control group. The intervention group had a lower incidence of heart failure or <10% drop in LVEF [30]. Patients who experienced troponin elevation after high-dose chemotherapy were randomized to be treated either with or without enalapril. None of the patients in the intervention group showed a decrease in LVEF [31].

In two meta-analyses of randomized clinical trials, angiotensin-converting enzyme inhibitors (ACEi)/angiotensin receptor blockers (ARB) or b-blocker (BB) treatment during chemotherapy in breast cancer patients proved beneficial. LVEF was preserved in patients on active prevention compared to placebo [32]. A 3.96% less decrease in LVEF was observed in patients receiving cardioprotective treatment [33].

In patients with an asymptomatic LVEF decrease, a cardio-oncology evaluation is recommended along with the commencement of cardioprotective agents (ACE-Is, ARBs, and/or BBs) and close monitoring [28]. Additionally, if LVEF drops under 40% or any symptom of heart failure presents, nonanthracycline chemotherapy is to be considered, and trastuzumab is to be ceased [34]. The rechallenge of trastuzumab should be decided after a multidisciplinary evaluation and discussion [28].

## 7. Long-Term Oncology Patient Follow-Up

Late-effect clinics are defined as cardio-oncology units that treat oncology patients long-term, especially those who have survived and have a high risk of or have already experienced cardiovascular complications. This mostly refers to adults who received chest radiation and are at a high risk of valvular, pericardial, or coronary artery disease; adults who survived from children or adolescent cancer; and patients who underwent a bone marrow transplant [22]. Concerning adult survivals of pediatric cancer, special monitoring is recommended for female survivors planning to become pregnant or during pregnancy and survivors planning high-intensity exercise (marathons, cycling, and triathlons) [8].

Cancer survivors who received cardiotoxic agents should be monitored for 6–12 months after the completion of treatment, 2 years later, and reassessed regularly. Several cardiovascular adverse events may appear in a later time period; therefore, a long-term follow-up is necessary for these patients. Valvular disease after chest radiotherapy may occur even 20 years after chemotherapy treatment, indicating the need for an echocardiogram every 5 years. Patients who experienced cardiotoxic events and received HF therapy should also be evaluated by a cardiologist as long as necessary. Additionally, long-term lifestyle modifications are recommended in order to benefit from recovery and avoid recurrence. Regular exercise for at least 150 min a week and a healthy balanced diet rich in fruits and vegetables could contribute to this purpose [28].

## 8. Limitations

This is a narrative review including limitations that need to be taken into consideration. First of all, cardio-oncology is a new field of cardiology, mostly developed within the last two decades. Due to the fact that there are still gaps in the literature regarding the frequency of cardiotoxic effects of chemotherapies in patients with malignancies, the evaluation of potential independent risk factors for cardiotoxicity, and the lack of widely approved and established follow-up protocols, we cannot extract safe conclusions but only suggestions on how to build and sustain a cardio-oncology clinic.

## 9. Conclusions

Cardio-oncology is rapidly growing field as the awareness about cardiovascular adverse events from cancer therapy is rising nowadays more than ever. As a result, more and more cardio-oncology programs are being established to surveil and treat cardiovascular manifestations before, during, and years after treatment in cancer patients. The operation of such programs faces many difficulties and requires the close collaboration of medical professionals from different specialties and staff from all ranks of governance. The increasing number of educational opportunities, the close guidance from professional organizations, such as ACC, AHA, ESC etc., the research opportunities, and the appropriate funding could provide the fuel to support and sustain the success of this program.
